# THE ROLE OF PEER HEALTH WORKS IN ENGAGING INCARCERATED PATIENTS IN RESEARCH

**DOI:** 10.1093/geroni/igac059.1681

**Published:** 2022-12-20

**Authors:** Fernando Murillo

**Affiliations:** University of California, San Francisco, San Francisco, California, United States

## Abstract

Incarcerated peers play a critical role in the provision of care for incarcerated older adults. Peer counsel is part of the culture in carceral settings and official and unofficial caregiving is a necessity for visually, mobility, and cognitively impaired residents of these institutions. Due to their experiences of exploitation by the prison system and research institutions, most older incarcerated people are reluctant to trust community partners or correctional staff members who conduct research in correctional settings. Many older residents who are offered participation in research do not believe any real systemic changes will occur with the publication of findings. This presentation will draw on autoethnographic experiences of a peer health worker in the palliative care setting. We will describe the role that peer health workers play in guiding patients in decision-making around research and interrogate a model of partnering with peer health workers to engage incarcerated older adults in research.

